# Evidence for Repeated Independent Evolution of Migration in the Largest Family of Bats

**DOI:** 10.1371/journal.pone.0007504

**Published:** 2009-10-21

**Authors:** Isabelle-Anne Bisson, Kamran Safi, Richard A. Holland

**Affiliations:** 1 Department of Ecology and Evolutionary Biology, Princeton University, Princeton, New Jersey, United States of America; 2 Max Planck Institute for Ornithology, Vogelwarte Radolfzell, Radolfzell, Germany; University of Bristol, United Kingdom

## Abstract

**Background:**

How migration evolved represents one of the most poignant questions in evolutionary biology. While studies on the evolution of migration in birds are well represented in the literature, migration in bats has received relatively little attention. Yet, more than 30 species of bats are known to migrate annually from breeding to non-breeding locations. Our study is the first to test hypotheses on the evolutionary history of migration in bats using a phylogenetic framework.

**Methods and Principal Findings:**

In addition to providing a review of bat migration in relation to existing hypotheses on the evolution of migration in birds, we use a previously published supertree to formulate and test hypotheses on the evolutionary history of migration in bats. Our results suggest that migration in bats has evolved independently in several lineages potentially as the need arises to track resources (food, roosting site) but not through a series of steps from short- to long-distance migrants, as has been suggested for birds. Moreover, our analyses do not indicate that migration is an ancestral state but has relatively recently evolved in bats. Our results also show that migration is significantly less likely to evolve in cave roosting bats than in tree roosting species.

**Conclusions and Significance:**

This is the first study to provide evidence that migration has evolved independently in bat lineages that are not closely related. If migration evolved as a need to track seasonal resources or seek adequate roosting sites, climate change may have a pivotal impact on bat migratory habits. Our study provides a strong framework for future research on the evolution of migration in chiropterans.

## Introduction

Why and how animals migrate represent quintessential questions in evolutionary biology. Birds and bats are the only flying animals to exhibit true seasonal return migration, broadly defined as the seasonal movements to and from breeding and non-breeding regions. For example, an estimated 30% of Palearctic bird species and as many as 45% of Nearctic species undergo seasonal migration [1]. More than a century of study has strived to discover the nature of these large-scale movements at a proximal level [reviewed in 2], [3]. Even more challenging has been the study of the evolution of migration. It is generally assumed that seasonal return migration evolves to allow animals to exploit seasonal abundance of food resources in higher latitudes, to avoid predators and to avoid disease [4], [1], [5]. Although studies on the evolutionary history of avian migration are well represented in the literature [e.g.], [ 1], [5]–[7], studies on migratory behavior in bats are relatively underrepresented [8].

Bats (Mammalia: Chiroptera) are the only mammals to have evolved true flight. Therefore bats have the potential to exploit seasonal abundance of food in temperate regions but migration is far less common than in birds. Less than 3% of extant bat species show migratory movement of any kind (for simplicity we define migration here as seasonal movements of greater than 50 km) with less than 0.016% of extant bat species moving over 1000 km in a one-way journey [8]. The majority of temperate bats undergo hibernation in the winter [10]. Nevertheless, migratory behavior has evolved in bats but to our knowledge, no hypotheses currently exist to explain how and when migration evolved in these animals. Interestingly, most [Bibr pone.0007504-Paradis1] [23 of 32] of the migratory species belong to the family Vespertilionidae, which are exclusively insectivorous. Long-distance migration appears to be correlated with tree roosting in temperate bats [11]. Given that such a large percentage of extant bats are tropical, it is possible that temperate bats evolved from tropical species. If this is the case then like birds they must have dispersed into the temperate zone to exploit new resources [7]. In birds this is thought to have driven the evolution of migration [1], [5], [6]. In bats however hibernation strategies are more common amongst temperate species, with only a few species wintering in the tropics (or sub tropical temperate regions). Indeed even long-distance migrants may hibernate once their wintering roosting area is reached [14]. Interestingly, a recent study suggests a Laurasian origin for bats, possibly in North America during the early Paleocene [15]. If this is indeed the case, it is possible that migration in modern temperate species evolved out of the northern hemisphere as a strategy to avoid the subsequent decreasing temperatures of northern climes. This scenario would have migratory behavior evolving at an early stage in the evolutionary history of bats. Alternatively, migration may have evolved out of the present day tropics as species expanded their range northward in order to track and exploit seasonal resources following Pleistocene glacial retreats as has been suggested for birds [1], [5], [6]. However, both explanations are not mutually exclusive. Migration may have evolved repeatedly and independently in the temperate zone and later in the tropics as multiple speciation events potentially occurred in tropical regions following northern climate change. In this study, we present a first analysis of the evolutionary history of migration in bats using a phylogenetic framework.

In the past it has not been possible to reconstruct the ancestral state of migration in bats as there was no existing phylogenetic “supertree” of bat species. However, Jones et al. [16] have recently presented a revised analysis of the first phylogenetic supertree [17] that includes 916 extant and nine extinct bat species. It is thus now possible to propose a workable model for the evolution of bat migration. The aim of this paper is to test hypotheses for the evolution of migration in bats using the Jones et al. [16] supertree. In light of current theories on the evolution of migration in birds [e.g., 5] and putative North American origins for bats, we use ancestral reconstruction methods to investigate if 1) migration evolved in bats as it is proposed for birds, and 2) whether migration may have appeared early in the evolutionary history of bats or whether it is a recently evolved trait.

## Methods

### Study system

Because most migratory bat species belong to the Vespertilionidae family, we used the Vespertilionidae supertree revised by Jones et al. [16] as a basis to reconstruct the evolutionary history of migration exclusively among species of this family. The high number of species (316) in the tree provides sufficient analytical power (phylogenetic signal) to formulate hypotheses on the evolution of migration in bats [18]. Phylogenetic relationships among species in Jones et al. [16] are based on a previously published species-level supertree of extant bat species [17], which incorporates family-level changes suggested by a recent molecular phylogenetic analysis by Teeling et al. [15]. The Jones et al. [16] supertree therefore represents a congruent one drawn from both molecular and morphological phylogenetic estimates. The monophyly of Vespertilionidae, although disputed in other works [19], [20], is well supported in the Jones et al. [16] supertree and in a recent molecular phylogenetic analysis [15]. Furthermore, lineages with migratory species such as *Lasiurus* were well resolved in the original Jones et al. [17] supertree with the exception of *L. egregious*. However, the monophyly of many genera that contained migratory species, are still unresolved such as *Pipistrellus* and *Lasiurus* with the exception of *Myotis* for which the monophyly is well supported.

The Jones et al. supertree [16] uses the taxonomic naming convention of Wilson and Reeder [21]. Currently there is no published comprehensive phylogeny that uses an updated taxonomic convention. Consequently, we adopted and changed taxonomic names to match the collated information on migration to the taxonomic names of the species used in the phylogeny. Therefore we also refer to the species in our study using the taxonomic convention of Wilson and Reeder [21], which meant that recently recognized species mainly in the genus *Pipistrellus* and *Plecotus* such as the *Pipistrellus pygmaeus* and *Plecotus kolumbatovici*, *P. alpinus* were assigned back to the species from which they were derived after the 1993 naming convention. Also the New-World long-eared bats and pipistrelles, which belong to the genus *Corynorhinus* and *Perimyotis* were renamed to *Plecotus* and *Pipistrellus*, and *Hypsugo savii* is being referred to as *Pipistrellus savii* according to the Wilson and Reeder [21] list of synonyms. It is important to realize that the changes in naming convention do not alter the position of a species on the phylogeny; it only changes the name with which a species is being attributed and thus these changes have no effect on the outcome of models. In other words, if a recent publication reports *Perimyotis subflavus* to be a short-distance migratory vespertilionid bat, the changes in naming convention will allow us to assign the migratory attribute to *Pipistrellus subflavus* in the phylogeny, which is the name of the species under which it is being referred to in the phylogeny.

### Ancestral state reconstruction and assumption testing

**Figure 1:**
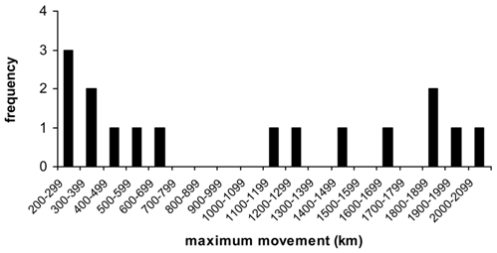
Frequency of migrants across a distance gradient in vespertilionid bats. This graph shows short-distance migrants generally move between 100 and 600 km and long-distance migrants moved between 1000 and 2000 km [8].

Migratory states were coded as non-migratory, short-distance migration, and long-distance migration according to the distinctions described in Fleming and Eby [8] but for European species this was cross-referenced with Hutterer et al. [9] (supplementary Table S1). Animal Diversity web [12] and Grzimek's Encyclopedia of Mammals [13] were subsequently checked for evidence of migration in species not contained in these two publications. In four cases only, (*Pipistrellus subflavus*, *Lasiurus seminolus*, *L. intermedius and L. ega*) the classification was based on personal communications (Lasiurines, P. Cryan, USGS, *P. subflavus*, A.C.Hicks, NYDEC). Maximum movement for the three *Lasiurine* species is unknown, but they were classified as long distance based on their relationship to the other migratory Lasiurine bats. *P. subflavus* was classified as a short distance migrant based on the personal comment of A. Hicks. Analyses were performed with and without the inclusion of these vespertilionids (see Results). Long-distance migrants are classified as moving more than 1000 km and short-distance migrants are classified as moving >100 km and <1000 km (data from [8], [9]). In practice short-distance migrants moved between 100 and 600 km and long-distance migrants moved between 1000 and 2000 km (Figure 1).

We used continuous-time Markovian models of trait evolution to model four hypotheses for the evolution of migration in bats. The continuous-time Markovian model is a probabilistic model that assumes, for any given time along the phylogenetic history, that migration state changes occur with a probability that depends only on the state of the immediately previous time step. That is, the future outcome of a process depends only on the present state and does not depend on when the last transition occurred or on the previous states. Since the continuous-time Markov chains are probabilistic models it is possible to calculate likelihood estimates of various models and use these values to test their validity by comparing their maximum likelihood [22], [23]. We fitted three standard models to the data and the phylogeny, known as the “equal”, “symmetrical”, and “all different” rate models and added a custom model assuming a three stage model of evolution. The “equal rates” model estimates one parameter of transition between all possible states. In the “symmetrical rates” model three parameters were estimated, which represent the symmetrical transition rates between all three states non-migratory, short-distance migration, and long-distance migration. In the “all rates different” model the transition rates of losses and gains between each combination of the three migratory states were assumed to be different and thus six different transition rates were estimated. Finally we specifically fitted a model, which represents the current bird model and assumes a three-stage model for the evolution of migration where one step is involved in a change (loss or gain) between the non-migratory/short-distance migratory states, one step between short-/long-distance migratory states, and two steps between the non-migratory/long-distance migratory states. The appropriate model was identified as the most parsimonious model, which reached convergence with the highest likelihood. To test for significant difference between the likelihood of two models we used twice the log likelihood difference, which follows a Chi-square distribution with degrees of freedom equal to the difference in numbers of estimated parameters [24]. In case of non-significant differences the model with the lower number of estimated parameters was preferred as the more parsimonious model. Thus, we determined the most likely evolutionary model for ancestral state reconstruction.

All analyses, plots, and tree alterations including random resolving of multichotomies were performed using R 2.8.1 [25] and the packages “Geiger” and “ape” [26], [27].

### Analysis of correlated trait evolution

We classified all migratory bats into one group to test whether migration might have evolved in a correlated fashion with roost use and/or geographic distribution (supplementary Table S1). Classifications came from a hierarchical search of Fleming and Eby [8], Hutterer et al. [9], Animal diversity web [12] and Grizimek's Encyclopedia of Mammals [13]. If the required information was not available in any of these publications the trait was classified as unknown. We first tested whether migration and roost type correlated without accounting for phylogenetic inertia using binomial logistic regression. Then we assessed whether the residuals of the model showed signs of phylogenetic non-independence using Moran's I and the phylogenetic distances of the species and whether the model estimates might have been flawed by the phylogenetic relationship between the species [28]–[30]. Using phylogenetic eigenvectors and the methods suggested by Diniz-Filho and colleagues [29] and the later extensions suggested by Desdevises et al. [31] we ran the same binomial logistic regression (phylogenetic eigenvector regression) to test for correlated trait evolution between migratory behavior and roost use while taking phylogenetic inertia into account.

## Results

A total of 23 species are known to exhibit migratory behavior (11 long-distance and 12 short-distance migrants) in the family Vespertilionidae (supplementary Table S1). The “symmetrical” and the “all rates different” models did not converge, indicating that the likelihood surfaces of these two models were flat and thus they did not fit the data [23]. Although not significantly different in their likelihood, the “equal rates” model represented the single best model compared to the three-stage model, as the more parsimonious model with the largest likelihood (equal rates model likelihood: −91.8, q±SE = 0.004±0.0006; 3 stage model likelihood: −93.2, q_0<->1_±SE = 0.008±0.0013, q_1<->2_±SE = 0.27±0.19). We repeated the analyses excluding four vespertilionids with an uncertain migratory status (*Pipstrellus subflavus*, and the Lasiurines *L. seminolus*, *L. ega and L. intermedius*). But a reanalysis of the data while removing them did not change the outcome of the study qualitatively (data not shown) and we present the results as they are for all the species and the original analysis.

**Figure 2:**
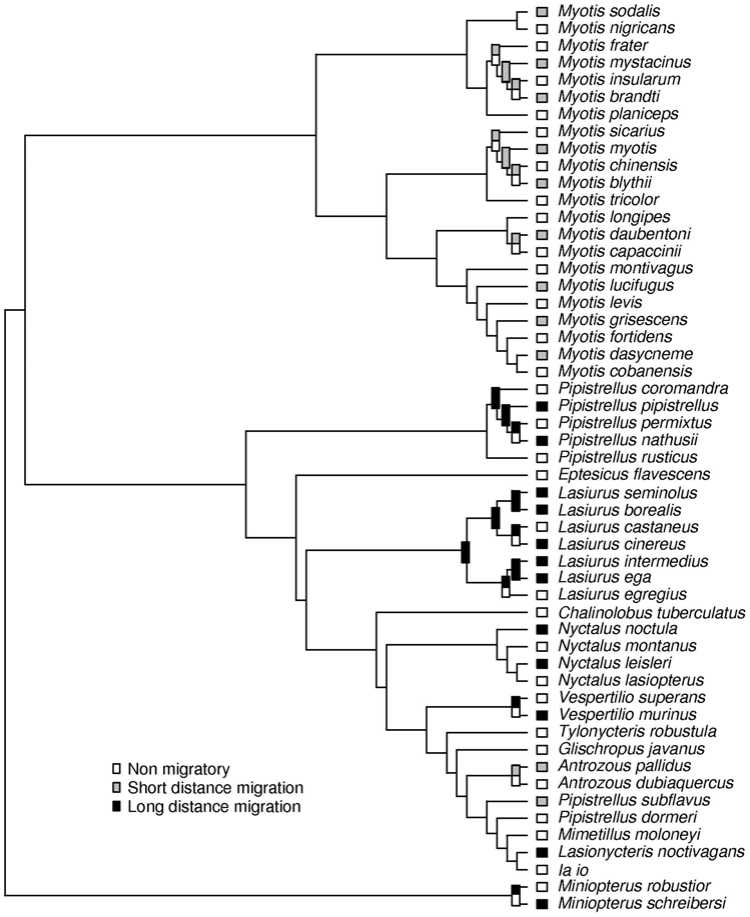
Ancestral state reconstruction of migratory behavior in vespertilionid bats. We used an equal rates Markovian model to reconstruct the evolutionary history of migration in the family Vespertilionidae. The tree shows the probabilities for migratory behavior for the internal branches for portions of the tree. The model and the ancestral states estimates were performed on the whole phylogeny but for better visibility we show only those species which display migratory behavior including two next sister taxa, as well as all the internal nodes and descendants which have reconstructed ancestral probabilities of more than 5% either for short- or long-distance migration. All nodes that have no assignment were classified as non-migratory with probabilities of more than 95%. The common ancestor for *P. nathusii*, *pipistrellus*, and *permixtus* is reconstructed as long-distance migrant as is the common ancestor for *Lasiurus cinereus*, *L. borealis*, *L. seminolus*, *L. castaneus*, *L. ega*, and *L. intermedius*.

Our analyses generally revealed that migratory behavior (short- and long-distance migration) evolved repeatedly and, for the most part, independently in vespertilionid bats (Figure 2). Exceptions include six species in the *Lasiurus* clade (Figure 2) and arguably two *Pipistrellus* species (*P. nathusii* and *P. pipistrellus*). Furthermore, long-distance migration appears to have evolved independently from short-distance migration, in most cases both migratory systems appear to have evolved from a sedentary ancestry suggesting that migration did not evolve from short- to long-distance migration. For example, short-distance migration is the only migratory system that has evolved in the *Myotis* clade and only two other species are short-distance migrants in the bats, *Pipistrellus subflavus* and *Antrozous pallidus*, without any evidence of long-distance migrant closely related taxa for these species. Moreover, most migratory vespertilionid species are temperate zone species.

The species level analysis suggested a significant correlation between migration and roost use as well as between migration and geographic distribution (Table 1). The effects of the interaction terms between roost use and geographic distribution on migratory behavior were non-significant and thus not entered in the final model (significance level for all interactions ≫0.05). In addition, the residuals of the species level analysis showed no significant level of phylogenetic autocorrelation (Moran's *I*: observed = −0.001±0.009, expected = −0.003, *P* = 0.83), which suggests that the estimates of the factors are not, or slightly flawed by phylogenetic inertia. Accordingly, after still taking the phylogenetic inertia into account, the model showed the same overall results of significantly lower likelihood of migration for tropical bats and those that roost in caves (Table 1). The phylogenetic eigenvector regression successfully removed the phylogenetic inertia from the model according to the Moran's I test for phylogenetic dependence among the residuals (Moran's *I*: observed = −0.003±0.0008, expected = 0.003, *P* = 0.9).

**Table 1:** Binomial logistic regression analysis at the species level and taking phylogenetic inertia into account.

	Species level model				Phylogenetically corrected model			
	Estimate	S.E.	z-value	P	Estimate	S.E.	z-value	P
Intercept	−5.1	1.0	−5.1	<0.0001	−6.5	1.8	−3.6	<0.0001
Temperate distribution	5.5	1.1	5.1	<0.0001	7.2	1.8	3.9	<0.0001
Cave	−1.3	0.6	−2.2	0.03	−3.1	1.1	−2.9	0.004
Building	−17.7	1929.4	0	0.99	−17.6	1882	0	0.99

## Discussion

Our results show that migration in vespertillionid bats, as indeed in all bats, is a relatively rare phenomenon that appears to have evolved independently in several lineages. The equal rates transition model was the most parsimonious explanation, suggesting that loss and gain of non-migratory behavior, short-distance migration, and long-distance migration are all equally likely in bats (Figure 2). The fact that the majority of extant bat species are tropical suggests a tropical origin for current temperate long-distance migratory species, but the lack of intermediate forms in the same lineage argues against long-distance migration having evolved from sedentary through short-distance to long-distance migration. The models also corroborated this. For example, the *Lasiurus* genus includes only long-distance migrants and the *Myotis* genus only short-distance migrant species (Figure 2). Unlike in birds, in which there is a continuous spread of migration distances from short- through medium-distance to long-distance, bats appear to have two distinct distance groups for migration, with a separation between the two (Figure 1). This may represent two different functions for short- and long-distance migration in bats. Indeed, short-distance migrants may have evolved from previously sedentary temperate hibernating species, whereas long-distance migrants, particularly the *Lasiurines*, may have evolved from tropical lineages.

There was no evidence for a migratory ancestral state in bats further supporting the hypothesis that migration evolved independently as a strategy to exploit seasonal resources, acquire higher quality hibernating and/or breeding habitat, and to potentially avoid predators or disease. Indeed migratory behavior in bats, as is suggested for birds [32], [33], may be far more plastic than originally believed. Migration in birds, particularly partial migration, where avian populations are composed of both migrant and resident individuals, is thought to be a polygenic and quantitative trait and may therefore be strongly influenced by environmental and social factors [34], [35]. In this context, the evolution and even the loss of migration in bats could be the outcome of rapid evolutionary changes in response to specific proximal factors. Although both birds and bats have evolved migration, the genetics of bat migration may be different to that of birds and migration strategies may have not appear as rapidly as they do in birds.

Although they represent the large majority of bat species, relatively little is known about the behavior of tropical and subtropical species. Even in the Lasiurines, their migratory behavior is inferred from seasonal distribution changes and stable isotope studies [36], [37] rather than from direct observation of migratory movements. Given that a recent paper has suggested that bats had a Laurasian origin, possibly in North America [15], it raises the intriguing possibility that the evolution of migration in vespertilionid bats was a response to falling temperatures and a retreat to warmer wintering climes rather than the “expansion from the tropics” that is generally viewed as the case in birds. Given that both long and short-distance migrants may hibernate when they reach their winter roost, the distance of migration may be influenced by the roosting ecology. Fleming and Eby [8] have noted that long-distance migrants are tree roosting species, whereas short-distance migrants are cave or building roosting species. Our study showed that tree-roosting bats are more likely to have evolved migration when compared to cave or building-roosting bats. It is possible that tree roosting species were forced to retreat farther south than cave roosting species to find suitable hibernating conditions. Moreover, Teeling et al. [15] further propose that the Vespertilionoidea microbat lineage evolved later during the Eocene epoch (50–52 Mya) when the planet was experiencing a 7°C temperature increase and an explosion in insect diversity. It is thus very possible that migration indeed evolved as a response to resource exploitation outside of regions experiencing a rapid increase in bat density. It should finally be noted that as well as temperate zone migrants in the Vespertilionidae, many of the other migrating bat species are tropical or sub-tropical [8]. In many cases it appears that these species migrate in response to seasonal distribution of fruiting trees. Their migration system appears very different to that of temperate zone migrants and it requires further analysis to determine how different migration strategies have evolved amongst the Chiroptera in a diverse array of ecosystems.

## Supporting Information

Table S1 Classification of character states for Vespertilionidae. We classified vespertilionid bats as either non-migratory (0), short distance migrant (1), and long distance migrant (2) according to the distinctions described in Fleming and Eby (8). Each species were further classified according to tropical (0) or temperate (1) species and roosting ecology (0 = tree, 1 = cave/building) as indicated by (8), by Animal Diversity Web (http://animaldiversity.ummz.umich.edu/site/index.html), and by Grizimek's Encyclopedia of Mammals.
(0.29 MB DOC)
