# Supplementary material for: Evidence for Repeated Independent Evolution of Migration in the Largest Family of Bats
Source: PLoS One. 2009 Oct 21;4(10):e7504. doi: 10.1371/journal.pone.0007504 (PMC2759287; doi:10.1371/journal.pone.0007504)
Supplement: Table S1 — Classification of character states for Vespertilionidae. We classified vespertilionid bats as either non-migratory (0), short distance migrant (1), and long distance migrant (2) according to the distinctions described in Fleming and Eby (8). Each species were further classified according to tropical (0) or temperate (1) species and roosting ecology (0 = tree, 1 = cave/building) as indicated by (8), by Animal Diversity Web (http://animaldiversity.ummz.umich.edu/site/index.html), and by Grizimek's Encyclopedia of Mammals. (0.29 MB DOC) [file pone.0007504.s001.doc]

| Species | Migration | Trop/temp | Roost |
| --- | --- | --- | --- |
| Miniopterus schreibersi | 2 | 1 | 1 |
| Miniopterus robustior | 0 | 0 | 1 |
| Miniopterus tristis | 0 | 0 | 1 |
| Miniopterus inflatus | 0 | 0 | 1 |
| Miniopterus magnater | 0 | ? | ? |
| Miniopterus fraterculus | 0 | 0 | 1 |
| Miniopterus fuscus | 0 | 0 | 1 |
| Miniopterus australis | 0 | 0 | 1 |
| Miniopterus minor | 0 | 0 | 1 |
| Miniopterus pusillus | 0 | 0 | 1 |
| Pipistrellus subflavus | 1 | 1 | 0 |
| Mimetillus moloneyi | 0 | 0 | 0 |
| Ia io | 0 | ? | ? |
| Eudiscopus denticulus | 0 | 0 | 0 |
| Philetor brachypterus | 0 | 0 | 0 |
| Otonycteris hemprichi | 0 | 0 | 1 |
| Nycticeius humeralis | 0 | 0 | 0 |
| Nycticeius rueppellii | 0 | 0 | 0 |
| Antrozous dubiaquercus | 0 | 0 | 1 |
| Antrozous pallidus | 1 | 0 | 0&1 |
| Myotis peninsularis | 0 | 0 | 1 |
| Myotis cobanensis | 0 | 0 | 1 |
| Myotis aelleni | 0 | 0 | 1 |
| Myotis abei | 0 | 0 | 1 |
| Myotis oxyotus | 0 | 0 | 1 |
| Myotis dasycneme | 1 | 0 | 1 |
| Myotis ricketti | 0 | 0 | 1 |
| Myotis volans | 0 | 1 | 1 |
| Myotis albescens | 0 | 0 | 1 |
| Myotis yumanensis | 0 | 0 | 1 |
| Myotis austroriparius | 0 | 0 | 1 |
| Myotis lucifugus | 1 | 1 | 1 |
| Myotis chiloensis | 0 | 0 | 1 |
| Myotis grisescens | 1 | 1 | 1 |
| Myotis fortidens | 0 | 0 | 1 |
| Myotis montivagus | 0 | 0 | 1 |
| Myotis levis | 0 | 0 | 1 |
| Myotis velifer | 0 | 0 | 1 |
| Myotis lesueuri | 0 | 0 | 1 |
| Myotis seabrai | 0 | 0 | 1 |
| Myotis macrotarsus | 0 | 0 | 1 |
| Myotis stalkeri | 0 | 0 | 1 |
| Myotis vivesi | 0 | 0 | 1 |
| Myotis riparius | 0 | 0 | 1 |
| Myotis simus | 0 | 0 | 1 |
| Myotis ruber | 0 | 0 | 1 |
| Myotis adversus | 0 | 0 | 1 |
| Myotis bocagei | 0 | 0 | 1 |
| Myotis hasseltii | 0 | 0 | 1 |
| Myotis horsfieldii | 0 | 0 | 1 |
| Myotis capaccinii | 0 | 1 | 1 |
| Myotis daubentoni | 1 | 1 | 1 |
| Myotis longipes | 0 | 0 | 1 |
| Myotis macrodactylus | 0 | 0 | 1 |
| Myotis pruinosus | 0 | 0 | 1 |
| Myotis formosus | 0 | 0 | 1 |
| Myotis welwitschii | 0 | 0 | 1 |
| Myotis emarginatus | 0 | 1 | 1 |
| Myotis goudoti | 0 | 0 | 1 |
| Myotis morrisi | 0 | 0 | 1 |
| Myotis tricolor | 0 | 0 | 1 |
| Myotis blythii | 1 | 1 | 1 |
| Myotis chinensis | 0 | 0 | 1 |
| Myotis myotis | 1 | 1 | 1 |
| Myotis sicarius | 0 | 0 | 1 |
| Myotis bechsteini | 0 | 1 | 1 |
| Myotis auriculus | 0 | 1 | 1 |
| Myotis evotis | 0 | 1 | 0 |
| Myotis keenii | 0 | 1 | 1 |
| Myotis milleri | 0 | 0 | 1 |
| Myotis pequinius | 0 | 0 | 1 |
| Myotis bombinus | 0 | 1 | 1 |
| Myotis nattereri | 0 | 1 | 1 |
| Myotis schaubi | 0 | 1 | 1 |
| Myotis thysanodes | 0 | 1 | 1 |
| Myotis siligorensis | 0 | 0 | 1 |
| Myotis scotti | 0 | 0 | 1 |
| Myotis oreias | 0 | 0 | 1 |
| Myotis altarium | 0 | 0 | 1 |
| Myotis californicus | 0 | 1 | 1 |
| Myotis leibii | 0 | 1 | 1 |
| Myotis planiceps | 0 | 0 | 1 |
| Myotis brandti | 1 | 1 | 1 |
| Myotis insularum | 0 | 0 | 1 |
| Myotis mystacinus | 1 | 1 | 1 |
| Myotis frater | 0 | 1 | 1 |
| Myotis annectans | 0 | 0 | 1 |
| Myotis australis | 0 | 0 | 1 |
| Myotis hosonoi | 0 | 0 | 1 |
| Myotis ikonnikovi | 0 | 0 | 1 |
| Myotis muricola | 0 | 0 | 1 |
| Myotis ozensis | 0 | 0 | 1 |
| Myotis ridleyi | 0 | 0 | 1 |
| Myotis rosseti | 0 | 0 | 1 |
| Myotis yesoensis | 0 | 0 | 1 |
| Myotis atacamensis | 0 | 0 | 1 |
| Myotis dominicensis | 0 | 0 | 1 |
| Myotis elegans | 0 | 0 | 1 |
| Myotis findleyi | 0 | 0 | 1 |
| Myotis keaysi | 0 | 0 | 1 |
| Myotis martiniquensis | 0 | 0 | 1 |
| Myotis nesopolus | 0 | 1 | 1 |
| Myotis nigricans | 0 | 0 | 1 |
| Myotis sodalis | 1 | 1 | 0 |
| Lasionycteris noctivagans | 2 | 1 | 0 |
| Tylonycteris pachypus | 0 | 0 | 0 |
| Tylonycteris robustula | 0 | 0 | 0 |
| Glischropus tylopus | 0 | 0 | 0 |
| Glischropus javanus | 0 | 0 | 0 |
| Harpiocephalus harpia | 0 | 0 | 0 |
| Murina grisea | 0 | 0 | 1 |
| Murina aenea | 0 | 0 | 1 |
| Murina cyclotis | 0 | 0 | 1 |
| Murina huttoni | 0 | 0 | 1 |
| Murina puta | 0 | 0 | 1 |
| Murina rozendaali | 0 | 0 | 1 |
| Murina aurata | 0 | 1 | 1 |
| Murina florium | 0 | 0 | 1 |
| Murina silvatica | 0 | 0 | 1 |
| Murina suilla | 0 | 0 | 1 |
| Murina tenebrosa | 0 | 0 | 1 |
| Murina tubinaris | 0 | 0 | 1 |
| Murina ussuriensis | 0 | 0 | 1 |
| Murina fusca | 0 | 0 | 1 |
| Murina leucogaster | 0 | 0 | 1 |
| Kerivoula aerosa | 0 | 0 | 0 |
| Kerivoula atrox | 0 | 0 | 0 |
| Kerivoula jagori | 0 | 0 | 0 |
| Kerivoula papuensis | 0 | 0 | 0 |
| Kerivoula africana | 0 | 0 | 0 |
| Kerivoula agnella | 0 | 0 | 0 |
| Kerivoula argentata | 0 | 0 | 0 |
| Kerivoula cuprosa | 0 | 0 | 0 |
| Kerivoula eriophora | 0 | 0 | 0 |
| Kerivoula flora | 0 | 0 | 0 |
| Kerivoula hardwickei | 0 | 0 | 0 |
| Kerivoula intermedia | 0 | 0 | 0 |
| Kerivoula lanosa | 0 | 0 | 0 |
| Kerivoula minuta | 0 | 0 | 0 |
| Kerivoula muscina | 0 | 0 | 0 |
| Kerivoula myrella | 0 | 0 | 0 |
| Kerivoula papillosa | 0 | 0 | 0 |
| Kerivoula pellucida | 0 | 0 | 0 |
| Kerivoula phalaena | 0 | 0 | 0 |
| Kerivoula picta | 0 | 0 | 0 |
| Kerivoula smithi | 0 | 0 | 0 |
| Kerivoula whiteheadi | 0 | 0 | 0 |
| Vespertilio murinus | 2 | 1 | 0 |
| Vespertilio superans | 0 | 1 | 0 |
| Laephotis wintoni | 0 | 0 | 0 |
| Laephotis namibensis | 0 | 0 | 0 |
| Laephotis botswanae | 0 | 0 | 0 |
| Laephotis angolensis | 0 | 0 | 0 |
| Histiotus macrotus | 0 | 0 | 0 |
| Histiotus velatus | 0 | 0 | 0 |
| Histiotus montanus | 0 | 0 | 0 |
| Histiotus alienus | 0 | 0 | 0 |
| Hesperoptenus doriae | 0 | 0 | 0 |
| Hesperoptenus blanfordi | 0 | 0 | 0 |
| Hesperoptenus gaskelli | 0 | 0 | 0 |
| Hesperoptenus tickelli | 0 | 0 | 0 |
| Hesperoptenus tomesi | 0 | 0 | 0 |
| Chalinolobus gouldii | 0 | 0 | 0 |
| Chalinolobus dwyeri | 0 | 0 | 0 |
| Chalinolobus morio | 0 | 0 | 0 |
| Chalinolobus nigrogriseus | 0 | 0 | 0 |
| Chalinolobus picatus | 0 | 0 | 0 |
| Chalinolobus tuberculatus | 0 | 0 | 0 |
| Nyctalus noctula | 2 | 1 | 0 |
| Nyctalus lasiopterus | 0 | 1 | 0 |
| Nyctalus leisleri | 2 | 1 | 0 |
| Nyctalus aviator | 0 | 0 | 0 |
| Nyctalus azoreum | 0 | 1 | 0 |
| Nyctalus montanus | 0 | 0 | 0 |
| Nycticeius greyii | 0 | 0 | 0 |
| Nycticeius sanborni | 0 | ? | ? |
| Nycticeius balstoni | 0 | 0 | 0 |
| Nycticeius schlieffeni | 0 | 0 | 0 |
| Scotoecus pallidus | 0 | 0 | 1 |
| Scotoecus albofuscus | 0 | 0 | 1 |
| Scotoecus hirundo | 0 | 0 | 1 |
| Rhogeesa alleni | 0 | 0 | 0 |
| Rhogeesa genowaysi | 0 | 0 | 0 |
| Rhogeesa parvula | 0 | 0 | 0 |
| Rhogeesa tumida | 0 | 0 | 0 |
| Rhogeesa minutilla | 0 | 0 | 0 |
| Rhogeesa gracilis | 0 | 0 | 0 |
| Rhogeesa mira | 0 | 0 | 0 |
| Lasiurus ega | 2 | 1 | 0 |
| Lasiurus intermedius | 2 | 1 | 0 |
| Lasiurus egregius | 0 | 0 | 0 |
| Lasiurus cinereus | 2 | 1 | 0 |
| Lasiurus castaneus | 0 | 0 | 0 |
| Lasiurus borealis | 2 | 1 | 0 |
| Lasiurus seminolus | 2 | 1 | 0 |
| Chalinolobus poensis | 0 | 0 | 0 |
| Chalinolobus beatrix | 0 | 0 | 0 |
| Chalinolobus argentatus | 0 | 0 | 0 |
| Chalinolobus alboguttatus | 0 | 0 | 0 |
| Chalinolobus egeria | 0 | 0 | 0 |
| Chalinolobus kenyacola | 0 | 0 | 0 |
| Chalinolobus gleni | 0 | 0 | 0 |
| Chalinolobus superbus | 0 | 0 | 0 |
| Chalinolobus variegatus | 0 | 0 | 0 |
| Nyctophilus arnhemensis | 0 | 0 | 0 |
| Nyctophilus geoffroyi | 0 | 0 | 0 |
| Nyctophilus gouldi | 0 | 0 | 0 |
| Nyctophilus heran | 0 | 0 | 0 |
| Nyctophilus microdon | 0 | 0 | 0 |
| Nyctophilus microtis | 0 | 0 | 0 |
| Nyctophilus timoriensis | 0 | 0 | 0 |
| Nyctophilus walkeri | 0 | 0 | 0 |
| Pharotis imogene | 0 | 0 | 0 |
| Barbastella barbastellus | 0 | 1 | 0 |
| Barbastella leucomelas | 0 | 0 | 0 |
| Euderma maculatum | 0 | 1 | 1 |
| Idionycteris phyllotis | 0 | 1 | 1 |
| Plecotus austriacus | 0 | 1 | 1 |
| Plecotus teneriffae | 0 | 1 | 1 |
| Plecotus auritus | 0 | 1 | 1 |
| Plecotus taivanus | 0 | 0 | 1 |
| Plecotus rafinesquii | 0 | 1 | 1 |
| Plecotus mexicanus | 0 | 0 | 1 |
| Plecotus townsendii | 0 | 1 | 1 |
| Scotomanes emarginatus | 0 | 0 | 0 |
| Scotomanes ornatus | 0 | 0 | 0 |
| Scotophilus kuhlii | 0 | 0 | 1 |
| Scotophilus nigrita | 0 | 0 | 1 |
| Scotophilus viridis | 0 | 0 | 1 |
| Scotophilus borbonicus | 0 | 0 | 1 |
| Scotophilus celebensis | 0 | 0 | 1 |
| Scotophilus heathi | 0 | 0 | 1 |
| Scotophilus leucogaster | 0 | 0 | 1 |
| Scotophilus robustus | 0 | 0 | 1 |
| Scotophilus dinganii | 0 | 0 | 1 |
| Scotophilus nux | 0 | 0 | 1 |
| Eptesicus floweri | 0 | 0 | 0 |
| Eptesicus nasutus | 0 | 0 | 0 |
| Eptesicus bobrinskoi | 0 | 0 | 0 |
| Eptesicus nilssoni | 0 | 1 | 1 |
| Eptesicus diminutus | 0 | 0 | 0 |
| Eptesicus innoxius | 0 | 0 | 0 |
| Eptesicus demissus | 0 | 0 | 0 |
| Eptesicus pachyotis | 0 | 0 | 0 |
| Eptesicus tatei | 0 | 0 | 0 |
| Eptesicus bottae | 0 | 0 | 0 |
| Eptesicus kobayashii | 0 | 0 | 0 |
| Eptesicus hottentotus | 0 | 0 | 0 |
| Eptesicus guadeloupensis | 0 | 0 | 0 |
| Eptesicus brasiliensis | 0 | 0 | 0 |
| Eptesicus furinalis | 0 | 0 | 0 |
| Eptesicus platyops | 0 | 0 | 0 |
| Eptesicus serotinus | 0 | 0 | 2 |
| Eptesicus fuscus | 0 | 0 | 2 |
| Pipistrellus dormeri | 0 | 0 | 0 |
| Pipistrellus circumdatus | 0 | 0 | 0 |
| Pipistrellus cuprosus | 0 | ? | ? |
| Pipistrellus societatis | 0 | 0 | 0 |
| Pipistrellus tasmaniensis | 0 | 0 | 0 |
| Pipistrellus affinis | 0 | 0 | 0 |
| Pipistrellus mordax | 0 | 0 | 0 |
| Pipistrellus petersi | 0 | 0 | 0 |
| Eptesicus baverstocki | 0 | ? | ? |
| Eptesicus douglasorum | 0 | 0 | 1 |
| Eptesicus pumilus | 0 | 0 | 1 |
| Eptesicus sagittula | 0 | 0 | 1 |
| Eptesicus regulus | 0 | 0 | 1 |
| Eptesicus vulturnus | 0 | 0 | 1 |
| Eptesicus somalicus | 0 | 0 | 0 |
| Eptesicus capensis | 0 | 0 | 0 |
| Eptesicus guineensis | 0 | 0 | 0 |
| Eptesicus brunneus | 0 | 0 | 0 |
| Eptesicus melckorum | 0 | 0 | 0 |
| Eptesicus rendalli | 0 | 0 | 0 |
| Eptesicus flavescens | 0 | 0 | 0 |
| Eptesicus tenuipinnis | 0 | 0 | 0 |
| Pipistrellus stenopterus | 0 | 0 | 0 |
| Pipistrellus anthonyi | 0 | 0 | 0 |
| Pipistrellus joffrei | 0 | 0 | 0 |
| Pipistrellus hesperus | 0 | 1 | 1 |
| Pipistrellus musciculus | 0 | 0 | 0 |
| Pipistrellus anchietai | 0 | 0 | 0 |
| Pipistrellus eisentrauti | 0 | 0 | 0 |
| Pipistrellus kitcheneri | 0 | 0 | 0 |
| Pipistrellus lophurus | 0 | 0 | 0 |
| Pipistrellus pulveratus | 0 | 0 | 0 |
| Pipistrellus arabicus | 0 | 0 | 0 |
| Pipistrellus nanus | 0 | 0 | 0 |
| Pipistrellus imbricatus | 0 | 0 | 0 |
| Pipistrellus ariel | 0 | 0 | 0 |
| Pipistrellus bodenheimeri | 0 | 0 | 0 |
| Pipistrellus savii | 0 | 1 | 0 |
| Pipistrellus cadornae | 0 | 0 | 0 |
| Pipistrellus macrotis | 0 | 0 | 0 |
| Pipistrellus crassulus | 0 | 0 | 0 |
| Pipistrellus nanulus | 0 | 0 | 0 |
| Pipistrellus rueppelli | 0 | 0 | 0 |
| Pipistrellus minahassae | 0 | 0 | 0 |
| Pipistrellus ceylonicus | 0 | 0 | 0 |
| Pipistrellus aegyptius | 0 | 0 | 0 |
| Pipistrellus aero | 0 | 0 | 0 |
| Pipistrellus kuhlii | 0 | 1 | 1 |
| Pipistrellus inexspectatus | 0 | 0 | 0 |
| Pipistrellus maderensis | 0 | 1 | 0 |
| Pipistrellus rusticus | 0 | 0 | 0 |
| Pipistrellus nathusii | 2 | 1 | 0 |
| Pipistrellus pipistrellus | 2 | 1 | 0 |
| Pipistrellus permixtus | 0 | 0 | 0 |
| Pipistrellus mimus | 0 | 0 | 0 |
| Pipistrellus tenuis | 0 | 0 | 0 |
| Pipistrellus coromandra | 0 | 0 | 0 |
| Pipistrellus babu | 0 | 0 | 0 |
| Pipistrellus javanicus | 0 | 0 | 0 |
| Pipistrellus endoi | 0 | 0 | 0 |
| Pipistrellus paterculus | 0 | 0 | 0 |
| Pipistrellus peguensis | 0 | 0 | 0 |
